# Two Patients With Extremely Long Type 1 Diabetes Duration With Very Few Complications and Remaining Insulin Secretion

**DOI:** 10.1002/ccr3.72183

**Published:** 2026-02-28

**Authors:** Åke Sjöholm, Daniel Espes

**Affiliations:** ^1^ Department of Internal Medicine, Division of Endocrinology and Diabetology Region Gävleborg, Gävle Hospital, University of Gävle Gävle Sweden; ^2^ Science for Life Laboratory Uppsala University Uppsala Sweden

**Keywords:** angiopathy, late complications, pancreatic beta‐cell, type 1 diabetes

## Abstract

We present two patients with > 80 years with type 1 diabetes (T1D), but with minor complications. Their history and risk factors are characterized as are factors conferring resilience against angiopathy. Amazingly, both had clearly detectable C‐peptide. The underlying reasons for their benign phenotype could form the basis for novel treatment targets.

## 1 Introduction

Type 1 diabetes (T1D) is a common disease and predominantly strikes young people [1]. This also means that many of these patients will live with T1D for a very long time, some virtually their entire life.

T1D is strongly associated with a much‐increased risk of premature and serious vascular complications and early death [1]. While many studies have clearly shown that this risk can be mitigated or delayed by optimal control of angiopathic risk factors at group levels [2], extensive clinical experience has made it clear that—independently of risk factor control—there is a great heterogeneity among individual T1D patients for their propensity to develop angiopathy. The underlying reasons for this remain largely elusive, but the identification of such factors would be paramount in developing not only new biomarkers but also novel targets for pharmaceutical intervention affording vascular protection.

## 2 Case Presentations

### 2.1 Case 1

An 85‐year‐old man of Swedish ethnicity was diagnosed in 1943 (at age 4) with insulin‐requiring diabetes. His grandmother and a cousin also suffered from insulin‐treated diabetes.

He had smoked varying amounts of tobacco between 1959 and 1982 and uses very little alcohol. He is also diagnosed with prostatic cancer, an aortic valve stenosis, and is confined to a wheelchair after surgery. The patient has abdominal obesity with a body mass index (BMI) of 34.4 kg/m^2^.

Despite 82 years of diabetes duration, the patient's only known late complications are non‐proliferative diabetic retinopathy (since 2009) and albuminuria (microalbuminuria which as recently as 2023 progressed to moderate macroalbuminuria).

### 2.2 Diagnostic Assessment

In 1943, the patient was diagnosed with diabetes with classic symptoms (polydipsia, polyuria, weight loss) and a blood glucose level of 170 mg/dL (SI: 9.4 mmol/L [reference range 76–115 mg/dL; SI: 4.2–6.4 mmol/L]). Blood glycated hemoglobin (HbA_1c_) measurements did not yet exist.

During the early years, he suffered frequent and severe hypoglycemic episodes (plasma glucose ~10–30 mg/dL [SI: 0.6–1.8 mmol/L]) with pronounced neuroglucopenic symptoms and loss of consciousness. No mention of diabetic ketoacidosis was found in the available medical records. In 2017 the method of glucose measurements was changed to continuous glucose monitoring, which rapidly resulted in a dramatic improvement in glucose levels, substantially less glycemic fluctuations, and virtually no significant hypoglycemias.

His blood HbA_1c_ levels over the past 15 years were 6.3%–9% (SI: 45–75 mmol/mol [reference range 5%–6.4%; SI: 31–46 mmol/mol]). His kidney function is normal with a plasma creatinine level of 1.11 mg/dL (SI: 98 μmol/L [reference range 0.68–1.28 mg/dL; SI: 60–105 μmol/L]), but in recent years he developed modest albuminuria; his urinary albumin‐creatinine ratio currently varies between 265 and 442 mg/g (SI: 30–50 mg/mmol [reference range < 26 mg/g; SI: < 3 mg/mmol]).

Plasma fasting low‐density lipoprotein (LDL) cholesterol currently is 104 mg/dL (SI: 2.7 mmol/L [reference range 77–205 mg/dL; SI: 2.0–5.3 mmol/L]), plasma fasting high‐density lipoprotein (HDL) cholesterol 66 mg/dL (SI: 1.7 mmol/L [reference range 31–81 mg/dL; SI: 0.8–2.1 mmol/L]), plasma fasting triglycerides 97 mg/dL (SI: 1.1 mmol/L [reference range 40–230 mg/dL; SI: 0.45–2.6 mmol/L]), and blood pressure usually 110–120/65–75 mmHg.

His current plasma C‐peptide level based on the clinical assay (both fasting and meal‐stimulated) is < 0.30 ng/mL (SI: < 0.10 nmol/L [reference range 1.1–4.5 ng/mL; SI: 0.37–1.5 nmol/L]). Plasma proinsulin is < 0.0035 pg/mL (SI: < 3.3 pmol/L [reference range 0.0035–0.03 pg/mL; SI: 3.3–28 pmol/L]). Insulin is detectable in plasma, as expected, reflecting the exogenous insulin therapy given. Antibodies against β‐cell autoantigens (glutamic acid decarboxylase 65‐kilodalton isoform [GAD65], islet antigen 2 [IA2], and zinc transporter 8 [ZnT8]) are negative.

### 2.3 Treatment

At diagnosis of diabetes in 1943, he was treated with recrystallized bovine insulin twice or thrice daily and oftentimes had non‐fasting blood glucose levels > 260 mg/dL (SI: 14.4 mmol/L).

From 1966 onwards, the patient's insulin regimen was changed to twice a day intermediate duration semilente insulin.

From at least 1987 he was treated with variable doses of human isophane insulin twice daily and regular soluble human insulin. In 1996 the latter insulin was switched to the direct acting analog insulin lispro as meal‐time insulin and in 2019 the isophane insulin was replaced by once a day insulin glargine.

Later in life he was put on treatment for dyslipidemia in 2007 (atorvastatin) and hypertension (metoprolol and amlodipine).

His antidiabetic therapy currently consists of insulin glargine once daily and insulin lispro as meal‐time insulin (total daily insulin dose 0.67 U/kg).

### 2.4 Outcome and Follow‐Up

We also measured the patient's plasma C‐peptide levels using an ultrasensitive human C‐peptide assay (Mercodia, Cat. No. 10‐1141‐01), which showed detectable C‐peptide at 0.0068 ng/mL (SI: 15.9 pmol/L). The ultrasensitive assay's lower detection limit is 0.0011 ng/mL (SI: 2.5 pmol/L), i.e., ~40 times more sensitive than conventional C‐peptide assays. Importantly, there is no cross‐reactivity at all to human insulin or any of the insulin analogs in clinical use.

Molecular genetic analysis for monogenic forms of diabetes was done by exome sequencing of his DNA against a panel of 54 known genes connected to monogenic diabetes (See Data S1); however, no pathogenic mutations were found.

Analysis of the patient's “T1D Genetic Risk Score” [3] was kindly done by Prof. A.T. Hattersley, Exeter, United Kingdom and was found to be equivalent to the 21st percentile of T1D patients, indicating that this patient is slightly more likely to have T1D than to have non‐T1D. Typing of human leucocyte antigens (HLA) showed that the patient lacks the DR3 haplotype (DRB1*03:01‐DQA1*05:01‐DQB1*02:01) but expresses the DR4 haplotype (DRB1*04:01‐DQA1*03:01‐DQB1*03:02), the latter predisposing to T1D.

Samples of his blood are stored frozen at −70°C pending potential future genetic analyses.

## 3 Case 2

An 87‐year‐old woman born in Sweden was diagnosed in 1941 (at age 3) with insulin‐requiring diabetes. Her grandmother and a cousin also suffered from insulin‐treated diabetes.

She has never used tobacco or alcohol in all her life. She has always been meticulous with her insulin treatment and her attempts to achieve good glucose control. Despite 84 years of diabetes duration, the patient's only known late complication is mild to moderate diabetic retinopathy.

### 3.1 Diagnostic Assessment

Her HbA_1c_ levels over the last 15 years were 5.2%–6.6% (SI: 33–49 mmol/mol). There is no microalbuminuria and her kidney function remains normal with a plasma creatinine level of 0.83 mg/dL (SI: 73 μmol/L). The patient is lean (BMI 22.3 kg/m^2^). Her plasma C‐peptide level based on the clinical assay (both fasting and meal‐stimulated) is < 0.30 ng/mL (SI: < 0.10 nmol/L). Plasma proinsulin is < 0.0035 pg/mL (SI: < 3.3 pmol/L). Insulin is detectable in plasma, as expected, reflecting the exogenous insulin therapy given. Antibodies against GAD65, IA2, and ZnT8 are negative.

Plasma fasting LDL cholesterol currently is 85 mg/dL (SI: 2.2 mmol/L), plasma fasting HDL cholesterol 70 mg/dL (SI: 1.8 mmol/L), plasma fasting triglycerides 97 mg/dL (SI: 1.1 mmol/L), and blood pressure usually 125–135/65–75 mmHg.

### 3.2 Treatment

The patient's initial insulin therapy consisted of long‐acting recrystallized bovine insulin once daily and short‐acting zinc‐insulin twice daily. Interestingly, during a couple of years in the early 1960s, chlorpropamide was added as adjunct therapy. However, when she stopped this due to hypoglycemia, a clear deterioration of glycemic control resulted, suggesting that she may at that time (~20 years after diagnosis) have had some residual insulin production. Her current antidiabetic therapy consists of human isophane insulin twice daily and insulin lispro as meal‐time insulin (total daily insulin dose 0.44 U/kg). In 2007, she started antihypertensive treatment (enalapril), and in 2009 also lipid lowering therapy (simvastatin).

### 3.3 Outcome and Follow‐Up

We also measured the patient's plasma C‐peptide levels using the ultrasensitive human C‐peptide assay described for patient 1, which showed detectable C‐peptide at 0.0073 ng/mL (SI: 17.17 pmol/L).

Molecular genetic analysis for monogenic forms of diabetes was done by exome sequencing of her DNA against a panel of 54 known genes connected to monogenic diabetes (Data S1); however, no pathogenic mutations were found. Analysis of the patient's “T1D Genetic Risk Score” [3] showed it to be equivalent to the 74th percentile of T1D patients, consistent with a diagnosis of T1D. HLA typing showed that the patient expresses both the DR3 haplotype (DRB1*03:01‐DQA1*05:01‐DQB1*02:01) and the DR4 haplotype (DRB1*04:01‐DQA1*03:01‐DQB1*03:02), both of which predispose to T1D.

Remarkably, during these 84 years she suffered only one severe hypoglycemic episode in the mid‐1950s. No records of diabetic ketoacidosis were found in the surviving documents. Curiously, the patient has not developed any unawareness of hypoglycemia despite her ultra‐long diabetes duration.

Samples of her blood are stored frozen at −70°C pending potential future genetic analyses.

## 4 Discussion

After the discovery of insulin in 1921, T1D was transformed from a universally fatal disease to a chronic condition. However, mortality remained high. It is empirically well known that some T1D patients develop premature and serious angiopathy despite excellent risk factor control, and *vice versa*, which begs the question: Why is that? The identification of such factors may aid in finding new biomarkers for vasculopathy and novel targets amenable to therapeutic intervention, benefiting the entire T1D population.

Patients with ultra‐long T1D duration and minimal complications are rare but may reveal key insights into mechanisms of disease resistance. The Joslin Diabetes Center Medalist Program introduced the 80‐year medal in 2013 and has since awarded not more than 19 such medals.

What, then, is it that makes these individuals survive without major complications for so long? Not surprisingly, this has been an area of intense research efforts for many years (see excellent and comprehensive reviews in [4, 5, 6]).

One obvious issue that must be addressed is the diagnostic considerations: Did these patients really have T1D, or were other forms of diabetes—less prone to complications—misdiagnosed as T1D? Many of the patients were diagnosed solely on clinical characteristics since this was long before autoantibody and C‐peptide analyses became available. To address this question, we conducted a molecular genetic analysis in order to rule out monogenic diabetes in our two patients, which turned out negative. Next, in order to sharpen the accuracy of a “true” T1D diagnosis, we investigated their HLA haplotypes, which, together with their “T1D Genetic Risk Score”, make T1D very probable. Astonishingly, however, they both had clearly detectable C‐peptide levels measured with an ultrasensitive assay despite > 80 years duration of T1D. Hence, we can conclude that the two investigated patients can be deemed to have T1D both based on their clinical presentation at childhood and based on modern diagnostic tools.

The canonical notion that T1D is a disease in which complete loss of β‐cells produces total insulin deficiency no longer holds true and we now know that there is residual β‐cell function in a fair share of patients even with > 50 years of T1D duration [7, 8, 9, 10], suggesting that some β‐cells for unknown reasons manage to evade the autoimmune attack. This implies that many patients with very long T1D duration may be “microsecretors” of insulin, and raises the important question whether their β‐cells really are dead or just non‐functional (“sleeping” [9, 10, 11]).

We could not detect any autoantibodies against β‐cell antigens in either of our patients, which could mean that after > 80 years of T1D autoimmunity may have disappeared [12], or that the remaining β‐cells either have a phenotype not recognized by the immune system or that the sheer number of β‐cells is low enough to not give rise to a systemically detectable response. In addition, we also do not know whether they ever had autoantibodies in the first place.

Several studies have attempted to identify factors linked to longevity and protection against vascular complications in T1D [4, 5, 6, 13, 14, 15, 16, 17]. As mentioned above, many T1D patients retain insulin production for several decades, and it seems this residual β‐cell function is associated with fewer microvascular complications [7, 14]. High HDL cholesterol, low triglycerides, lack of albuminuria, and low insulin requirements are all linked to microvascular protection [4, 5, 6, 7, 13, 14, 15, 16, 17]. Notably, of our patients, one (case 2) did not even have microalbuminuria despite 84 years with diabetes, and the other with 81 years of diabetes (case 1) progressed to modest macroalbuminuria only very recently.

In T1D adults, the total daily insulin requirement usually is 0.5–1.0 U/kg [18]. As low insulin doses were found to be associated with vascular protection [4, 5, 6, 14], it is of interest to note that our two patients had fairly low insulin requirements, 0.67 U/kg/d (case 1) and 0.44 U/kg/d (case 2). Especially when considering the level of restriction for physical activity in case 1 due to the fact that he is confined to a wheelchair.

Ultra‐long T1D survivors provide insight into mechanisms of vascular protection that go beyond glycemic control alone. Studying these individuals may have great implications for future research in the search of (and may even be required for) new therapeutic targets and biomarkers for preventing both micro‐ and macrovascular complications in T1D.

## 5 Learning Points

- Factors associated with surviving > 80 years with type 1 diabetes with only minor complications are discussed.
- Insulin microsecretion may persist despite exceedingly long duration of type 1 diabetes.
- The identification of factors conferring β‐cell protection and resistance to angiopathy may reveal not only new biomarkers but also novel targets amenable to therapeutic intervention, benefiting the entire T1D population.

## Author Contributions

**Åke Sjöholm:** conceptualization, investigation, writing – original draft, writing – review and editing. **Daniel Espes:** methodology.

## Funding

The authors have nothing to report.

## Ethics Statement

IRB approval is not required in Sweden for the publication of case reports. Ethical guidelines were followed.

## Consent

Signed informed written consents were obtained directly from the patients.

## Conflicts of Interest

The authors declare no conflicts of interest.

## Supporting information


**Data S1:** ccr372183‐sup‐0001‐Supinfo1.docx.

## Data Availability

Restrictions apply to the availability of some or all data generated or analyzed during this study to preserve patient confidentiality or because they were used under license. The corresponding author will on request detail the restrictions and any conditions under which access to some data may be provided.
