# Supplementary material for: Two Patients With Extremely Long Type 1 Diabetes Duration With Very Few Complications and Remaining Insulin Secretion
Source: Clin Case Rep. 2026 Feb 28;14(3):e72183. doi: 10.1002/ccr3.72183 (PMC12949425; doi:10.1002/ccr3.72183)
Supplement: Supplementary file 1 — Data S1: ccr372183‐sup‐0001‐Supinfo1.docx. [file CCR3-14-e72183-s001.docx]

*Supplementary Material to:*

**Two Patients with Extremely Long Type 1 Diabetes Duration with Very Few Complications and Remaining Insulin Secretion**

**Åke Sjöholm, M.D., Ph.D. and Daniel Espes, M.D., Ph.D.**

Molecular genetic analysis for monogenic forms of diabetes

Whole exome sequencing of DNA was done at Karolinska University Laboratory against a panel of 54 known genes connected to monogenic diabetes (*ABCC8, AGPAT2, AIRE, AKT2, APPL1, BSCL2, CEL, CIDEC, CISD2, CNOT1, COQ2, COQ9, CTLA4, DCAF17, DNAJC3, DYRK1B, EIF2AK3, EIF2B1, FOXP3, GATA4, GATA6, GCK, GLIS3, HNF1A, HNF1B, HNF4A, IER3IP1, IL2RA, INS, INSR, ITCH, KCNJ11, LIPE, LMNA, MAFA, NEUROD1, NEUROG3, NKX2-2, PAX6, PCBD1, PDX1, PIK3R1, POLD1, PPARG, PPP1R15B, PTF1A, RFX6, SLC19A2, SLC29A3, SLC2A2, TRMT10A, WFS1, ZFP57, ZMPSTE24*).
